# Complete Genome Sequence of Serratia marcescens Siphophage Slocum

**DOI:** 10.1128/MRA.01421-19

**Published:** 2020-01-02

**Authors:** Jason Snowden, Heather Newkirk, Russell Moreland, Mei Liu, Jolene Ramsey, Justin Leavitt

**Affiliations:** a Center for Phage Technology, Texas A&M University, College Station, Texas, USA; Queens College

## Abstract

Serratia marcescens is a ubiquitous Gram-negative opportunistic pathogen. This announcement describes the isolation and genome annotation of S. marcescens T5-like siphophage Slocum. Terminal repeats, 170 protein-coding genes, and 23 tRNAs were predicted in the 112,436-bp Slocum genome.

## ANNOUNCEMENT

Serratia marcescens is a Gram-negative bacillus that is ubiquitous throughout nature and is a member of the family *Enterobacteriaceae* (1). S. marcescens causes infections in immunocompromised patients and infants and was recently reported as a pathogen of several bee species (2, 3). In this study, the S. marcescens*-*infecting bacteriophage Slocum was isolated and its genome was annotated.

Bacteriophage Slocum originated from filtered (0.2-μm filter) municipal wastewater in Bryan, Texas. The host, S. marcescens strain D1 (no. 8887172; Ward’s Science), was cultured aerobically at 30°C and 37°C in LB (BD), and phages were propagated via the soft-agar overlay method (4). After phages were stained with 2% (wt/vol) uranyl acetate, Slocum morphology was determined by transmission electron microscopy at the Texas A&M University Microscopy and Imaging Center (5). Phage genomic DNA was purified with the modified Promega Wizard DNA clean-up system shotgun library preparation protocol described by Summer (6), and then an Illumina TruSeq Nano low-throughput kit was used to prepare a library for Illumina MiSeq sequencing with paired-end 250-bp reads, using a 500-cycle v2 kit. Quality control of 2,820,474 total sequenced reads was performed using FastQC (http://www.bioinformatics.babraham.ac.uk/projects/fastqc). After trimming was performed using the FastX Toolkit v0.0.14 (http://hannonlab.cshl.edu/fastx_toolkit), the raw Slocum contig was assembled using SPAdes v3.5.0, with default parameters, to 409.3-fold coverage (7). PCR from the contig ends (forward, 5′-CCGTTGTTGCACAAGATGAAG-3′; reverse, 5′-ACTAGGGTATGCCTAAGAGGAAA-3′) and Sanger sequencing of the products were used to verify a complete and accurate sequence. Structural annotations relied on gene calling by MetaGeneAnnotator v1.0 and GLIMMER v3.0 for proteins and ARAGORN v2.36 for tRNAs (8–10). Rho-independent termination sites were annotated using TransTermHP v2.09 (11). Functional predictions of genes were made using InterProScan v5.33-72, TMHMM v2.0, and BLAST v2.2.31 with NCBI nonredundant and UniProtKB Swiss-Prot/TrEMBL databases, with a maximum expectation value of 0.001 (12–15). Additionally, coding sequences were analyzed for lipoylation signals with LipoP v1.0 (16). Structural predictions were performed with HHSuite v3.0 tool HHPred (multiple sequence alignment generation with HHblits using the ummiclus30_2018_08 database and modeling with the PDB_mmCIF70 database) (17). Genome-wide DNA sequence similarity for Slocum was calculated using progressiveMauve v2.4.0 (18). The genome termini were predicted with PhageTerm (19). All listed annotation tools and their outputs (with the exception of HHPred) are available in the Galaxy and Web Apollo instances hosted at the Center for Phage Technology (CPT) (https://cpt.tamu.edu/galaxy-pub) (20, 21). Unless otherwise stated, all tools were executed using default parameters.

The phage Slocum genome consists of 112,436 bp of double-stranded DNA, with a G+C content of 44.8%. With 170 predicted protein-coding genes and 23 predicted tRNA genes, the genome protein coding density is 88%. Additionally, 12,521-bp predicted direct terminal repeats were used as the boundary for genome reopening. At the amino acid level, phage Slocum shares 81 similar proteins with *Escherichia* phage T5 (GenBank accession no. NC_005859) and T5-like *Escherichia* phage slur09 (GenBank accession no. LN887948), with 27 to 29% nucleotide identity to slur09 and several *Salmonella* phages, fragmented across the genome.

### Data availability.

The genome sequence and associated data for phage Slocum were deposited under GenBank accession no. MN095770, BioProject accession no. PRJNA222858, SRA accession no. SRR8892204, and BioSample accession no. SAMN11411459.
